# Single-cell RNA sequencing reveals the molecular features of peripheral blood immune cells in children, adults and centenarians

**DOI:** 10.3389/fimmu.2022.1081889

**Published:** 2023-01-10

**Authors:** Jinjie Zhong, Rong Ding, Huimin Jiang, LongFei Li, Junli Wan, Xiaoqian Feng, Miaomiao Chen, Liping Peng, Xiaoqin Li, Jing Lin, Haiping Yang, Mo Wang, Qiu Li, Qilin Chen

**Affiliations:** ^1^ Department of Nephrology Children’s Hospital of Chongqing Medical University, Chongqing, China; ^2^ National Clinical Research Center for Child Health and Disorders, Ministry of Education Key Laboratory of Child Development and Disorders, Chongqing, China; ^3^ Chongqing Key Laboratory of Pediatrics, Chongqing, China; ^4^ Nanjing Jiangbei New Area Biopharmaceutical Public Service Platform Co. Ltd, Nanjing, Jiangsu, China

**Keywords:** single-cell RNA sequencing (scRNAseq), peripheral blood mononuclear cells, whole lifespan, autoimmune diseases, CD8^+^ cytotoxic T cells

## Abstract

Peripheral blood immune cells have different molecular characteristics at different stages of the whole lifespan. Knowledge of circulating immune cell types and states from children to centenarians remains incomplete. We profiled peripheral blood mononuclear cells (PBMCs) of multiple age groups with single-cell RNA sequencing (scRNA-seq), involving the age ranges of 1-12 (G1), 20-30(G2), 30-60(G3), 60-80(G4), and >110 years (G5). The proportion and states of myeloid cells change significantly from G1 to G2. We identified a novel CD8^+^CCR7^+^GZMB^+^ cytotoxic T cell subtype specific in G1, expressing naive and cytotoxic genes, and validated by flow cytometry. CD8^+^ T cells showed significant changes in the early stage (G1 to G2), while CD4^+^ T cells changed in the late stage (G4 to G5). Moreover, the intercellular crosstalk among PBMCs in G1 is very dynamic. Susceptibility genes for a variety of autoimmune diseases (AIDs) have different cell-specific expression localization, and the expression of susceptibility genes for AIDs changes with age. Notably, the CD3^+^ undefined T cells clearly expressed susceptibility genes for multiple AIDs, especially in G3. *ETS1* and *FLI1*, susceptibility genes associated with systemic lupus erythematosus, were differentially expressed in CD4^+^ and CD8^+^ effector cells in G1 and G3. These results provided a valuable basis for future research on the unique immune system of the whole lifespan and AIDs.

## Introduction

The evolution of robust and flexible defense responses has been driven by the diverse and adaptive nature of foreign threats. To maintain its effectiveness, the immune system produces highly specialized cell types that work together to prevent, preserve the memory and eliminate threats (1). Meanwhile, human immune system cells undergo time-dependent deterioration as they are continuously stimulated by internal and external factors (2). The aging adaptive immune system, characterized by genomic instability, telomere depletion, epigenetic alterations, and a loss of protein homeostasis, exhibits progressive dysfunction and increased autoimmunity (2, 3).

Unbiased single-cell techniques have been used to characterize homogeneous immune cell populations in health and disease, discover stochastic gene expression changes that drive immune responses, and reconstruct developmental trajectories for immune cells (4). Single-cell RNA sequencing (scRNA-seq) research of mouse and human CD8^+^ T cells revealed a surprising population of distinct age-related CD8^+^ T cells (5). Similarly, scRNA-seq from aged and young humans found that naive CD8^+^ T cells were significantly reduced in old age (6, 7). Combining the bulk and single-cell RNA profiles of lymphocytes, myeloid cells, and hematopoietic stem and progenitor cells from fetal, perinatal, and adult developmental stages, the team demonstrated that the transition of immune cells from the fetal period to adulthood occurs gradually along a maturation continuum, which they named progressive changes (8).

In addition, the degeneration of the thymus, successive stimulation of foreign neoantigens, and immaturity of the adaptive immune system shape the immune system in childhood, with characteristics that are different from those of other ages (9). Children’s unique innate and adaptive immune responses may be related to the “relative safety” in coronavirus disease 2019 (COVID-19) (10–12). By flow cytometry, previous studies have found that the percentage of naive cells decreased and the percentage of memory cells increased from age 0 to 18 years, regardless of whether it was CD4^+^ T cells or CD8^+^ T cells (13–15). However, scRNA-seq studies on childhood immune cells are still lacking. Incorporating childhood into the interpretation of immune system changes will contribute to understanding the development of and changes in the immune system over the lifespan and provide new insights into autoimmune diseases (AIDs) (16).

We constructed a peripheral blood mononuclear cells (PBMCs) profile involving the age ranges of 1-12, 20-30, 30-60, 60-80, and >110 years and comprehensively displayed the transcriptional characteristics of immune cells from childhood to old age at the single-cell level. We focused on the characteristics of myeloid cells, CD4^+^ T cells, CD8^+^ T cells and B cells in multiple age groups, and investigated the changes in crosstalk between cells. Finally, combined with the genome-wide association studies (GWAS) of AIDs, it was found that susceptibility genes for a variety of AIDs have different cell-specific expression localization, and the expression of susceptibility genes for AIDs changes with age. These results provide a valuable basis for future research on the immune system of the whole lifespan and AIDs.

## Methods

### Donors

The study was reviewed and approved by Institutional Review Board of Children’s Hospital of Chongqing Medical University. The ethics approval number for this study is 2022 Research 1. Written informed consent was obtained from all healthy donors or their guardians. We recruited 3 healthy children for scRNA-seq, while the additional 10 healthy children and 8 healthy adults were recruited for flow cytometry validation experiments. All donors were recruited from Children’s Hospital of Chongqing Medical University between January 2022 and June 2022. The inclusion criteria of healthy donors were as follows: children aged 1-12 years, adults aged 20-40 years, no gender restriction, without underlying diseases or the results of routine blood and urine tests were normal. For each donor, 2 ml of venous blood was collected in EDTA anticoagulant tubes and transferred to the laboratory on ice. PBMCs were isolated from whole blood by density gradient centrifugation using Ficoll medium (TBD, Tianjing, China). For 3 healthy children whose samples underwent scRNA-seq, PBMCs were frozen until analyzed according to the 10X genomics recommended protocol (CG00039). For 10 healthy children and 8 healthy adults whose samples underwent flow cytometry, PBMCs were processed immediately. In addition, we included 35 published healthy donors whose PBMCs underwent scRNA-seq using the 10X Genomics platform. Details about each sample and data can be found in **Supplementary Table 1**.

### scRNA-seq library construction and sequencing

PBMCs from 3 healthy childhood donors were thawed according to the 10X genomics recommended protocol (CG00039), and the cell viability of each sample was >80%. The PBMCs from each sample were diluted to a final concentration of 700~1200 cells/µl, and approximately 16000 cells per reaction were loaded on a Chromium Single Cell Controller (10X Genomics, Pleasanton, CA). The libraries for scRNA-seq were constructed using the Chromium Next GEM Single Cell 3’ GEM, Library & Gel Bead Kit v3.1 (10X Genomics, PN-1000121) following the manufacturer’s protocol. Libraries were sequenced on an Illumina NovaSeq 6000. Each sample was processed independently.

### scRNA-seq data processing

For 3 unpublished data, the raw sequencing reads were processed using Cell Ranger (version 6.0.0). The reference index was built using the GRCh38 human reference genome assembly. The dataset in this study includes 3 unpublished scRNA-seq data and 35 published public data from GSE168732, GSE158055 and the link provided by the article (http://gerg.gsc.riken.jp/SC2018/). Among them, the author of GSE158055 provided the data after quality control, so the quality control will not be repeated. Unpublished datasets, GSE168732 and SC2018 datasets are quality-controlled based on data characteristics. The quality standards include the number of genes in cells (nFeature), the number of UMIs in cells (nCount), and the distribution ratio of mitochondrial gene content in cells (Mitochondrion) (**Supplementary Table 3**). After removing unwanted cells from the dataset, the next step is to normalize the data using Seurat (version 4.0.5). By default, we employ a global-scaling normalization method “LogNormalize” that normalizes the feature expression measurements for each cell by the total expression, multiplies this by a scale factor (10,000 by default), and log-transforms the result. 38 samples were integrated using Seurat (version 4.0.5). Due to the large number of samples, the strategy of Reciprocal Principal Component Analysis (RPCA) and Reference-based integration were adopted. We observed samples from different datasets and found that batch effects between datasets have been removed. Principal component analysis (PCA) was performed on the highly variable genes. The graph-based clustering algorithm is used for clustering, which constructs a K-Nearest Neighbor (KNN) graph through Euclidean distance, and the Louvain algorithm is used to group cells and optimize modules. We applied KNN graph to unsupervised clustering of cells. T-distributed stochastic neighbor embedding (t-SNE) and uniform manifold approximation and projection (UMAP) algorithms were used to visualize clustered cells in 2D space.

### Correlation analysis

The gene expressions of single-cell level of samples were averaged and fitted to bulk-level, using the AverageExpression function of Seurat. Then we calculated the Pearson’s correlation coefficient between samples. In addition, PCA was applied to analyze the relationship between PC and sample distribution in different age groups.

### Differential gene expression and functional enrichment analysis

The comparison of gene expression between a certain cluster and other clusters was conducted by the FindMarkers function of Seurat using the Wilcoxon rank sum test to obtain the differentially expressed genes (DEGs) dataset of the certain cluster compared with other clusters (threshold: log (fold change) >= 0.25, *p* adjust value (Bonferroni correction) < 0.01). The DEGs datasets of a cluster in one group compared with the cluster in other groups (G1vsG2, G1vsG3, G1vsG4, G1vsG5) and a cluster compared with other clusters in the same group were built using the protocol described before (6). UpSetR (version 1.4.0) was used to build the specific DEGs datasets and common DEGs datasets by making intersections between different clusters or different groups. For example, in the DEGs datasets of G1vsG2, some DEGs only existed in myeloid cell and we named it “myeloid-specific DEGs” or “cell-specific DEGs”, while some DEGs existed in all cell types and we named it “common DEGs”. Enrichment analysis for the functions of the DEGs and Protein-protein Interaction enrichment analysis (PPI) were conducted using the Metascape webtool (www.metascape.org). Gene sets were derived from the Gene Ontology (GO) Biological Process ontology and Kyoto Encyclopedia of Genes and Genomes (KEGG) pathway.

### Defining cell function and state scores

We used the AddModuleScore function of Seurat to evaluate the expression degree of a certain predefined expression gene set. The cell scores were based on the average expression of the genes from the predefined gene set in the respective cell. We used immune effector process (GO:0002252), activation of immune response (GO:0002253), establishment or maintenance of cell polarity (GO:0007163), regulation of defense response (GO:0031347), leukocyte activation (GO:0045321), leukocyte homeostasis (GO:0001776), cytokine production involved in immune response (GO:0002367), leukocyte migration (GO:0050900), response to virus (GO:0009615), immunoglobulin mediated immune response (GO:0016064), regulation of mast cell activation involved in immune response (GO:0033006), 4 naive marker genes (*CCR7*, *TCF7*, *LEF1*, *SELL*), 12 cytotoxic marker genes (*PRF1*, *IFNG*, *GNLY*, *NKG7*, *GZMA*, *GZMB*, *GZMH*, *KLRK1*, *KLRB1*, *KLRD1*, *CTSW*, *CST7*) and 6 exhaustion marker genes (*TOX*, *PD1*, *LAG3*, *TIGIT*, *GZMK*, *CCL5*, *CTLA4*) to define some meaningful functions and states.

### Transcription factor analysis

We used pyscenic (version 0.11.2) to analyze the gene regulation network of scRNA-seq datasets.

### Ligand–receptor interaction analysis

Cellphone DB was used to analyze the crosstalk between cell clusters based on ligand–receptor relationships. The results were visualized using igraph (version 1.2.10).

### Pseudotime analysis

Monocle3 (version 1.0.0) was used to predict development trajectories. The umap datasets of Seurat were directly imported into Monocle3 for pseudotime analysis, with the start of the pseudotime set to the highest correction ratio in the G1 group.

### Analysis of GWAS gene expression

We downloaded the susceptibility gene sets of some immune-related diseases from the genome-wide association studies Catalog (https://www.ebi.ac.uk/gwas), including gene sets for Kawasaki disease, rheumatoid arthritis, Graves’ disease, type I diabetes, systemic lupus erythematosus and nephrotic syndrome. A gene heatmap was used to observe the expression in the PBMCs of this susceptibility gene set in different groups, and the Seurat AddModuleScore function was used to evaluate the degree of expression.

### Flow cytometric analysis

Flow cytometry was performed on the remaining blood samples of donor CON-2 and CON-3 after scRNA-seq samples were retained. Another cohort of 10 healthy children and 8 healthy adults was recruited and their blood samples were collected for flow cytometry. To evaluate the expression of T cell surface markers by flow cytometry, 200μL whole blood was incubated with the following antibodies, 7-AAD (BioLegend, 420406), CD3-Pacific Blue (BioLegend, 300330), CD4-PE/CY7 (Invitrogen™, 25-0049-42) and CD8-APC/CY7 (BioLegend, 344714). After staining for 20 minutes at room temperature in the dark, erythrocytes (BD Pharmingen, 555899) in the samples were lysed by incubation with lysing solution for 5 minutes. Following centrifugation (300g/5 minutes, 4℃) and washing with PBS, cells were then examined using BD FASCCanto™. For intracellular staining, PBMCs were cultured by RPMI-1640 medium containing 10%FBS, 50ng/ml PMA, 1ug/ml Streptomycin and 1μl/ml Golgi-stop in 37℃, 5% CO_2_ for 4h, and were dealt with Fixation/Permeabilization Solution (Invitrogen™,00-5523-00). Flow cytometry analysis for CCR7^+^GZMB^+^CD8^+^cytotoxic T cells was carried out in BD FASCCanto™ using the following antibodies: CD3-FITC (BioLegend,300452), CD4-PE/CY7 (Invitrogen™, 25-0049-42), CD8-APC/CY7 (BioLegend,344714), BV421-CCR7 (BioLegend,353207), PE-GZMB (BD Pharmingen, 56114). The datasets were analyzed using FlowJo (version 10.4.2).

### Statistical analysis

Flow cytometry analysis was performed using unpaired t-test by GraphPad Prism 8, and *P*<0.05 was considered statistically significant. For others, unless otherwise stated, all data were analyzed using the two-sided Wilcoxon rank sum test, and *P* < 0.01 was considered statistically significant.

## Results

### The single-cell profile of PBMCs in multiple age groups

**Figure 1:**
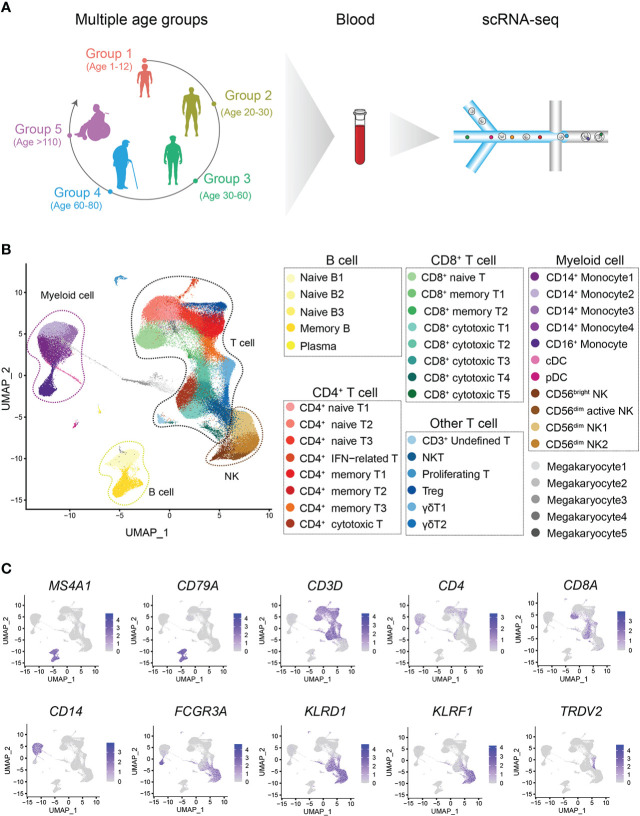
Single-cell transcriptome profiling of the PBMCs of healthy children and multiple other age groups. **(A)** Schematic representation of the cell profile of blood immune cells in multiple age groups. **(B)** Two-dimensional UMAP visualization of PBMCs for multiple age groups. Different colors represent 43 clusters (cell types). **(C)** Expression of marker genes for 7 main cell types; cell positions are from the UMAP plot in **(B)**.

We constructed a healthy multiage PBMC profile including children (n=6, 1-12 years old, Group 1, G1), young adults (n=8, 20-30 years old, Group 2, G2), middle-aged adults (n=12, 30-60 years old, Group 3, G3), aged adults (n=5, 60-80 years old, Group 4, G4) and supercentenarians (n=7, >110 years old, Group 5, G5) (**Figure 1A**). The basic profile of healthy donors in each age group is shown in **Supplementary Table 1**. After the unified single-cell analysis pipeline (Methods), 44689 cells (20.23%) were from children (G1), 42351 cells (19.17%) were from young adults (G2), 68153 cells (30.85%) were from middle-aged adults (G3), 32007 cells (14.49%) were from aged adults (G4), 33707 cells (15.26%) were from supercentenarians (G5). All high-quality cells (220907 cells) were integrated into an unbatched and comparable dataset and subjected to principal component analysis after correction for read depth and mitochondrial read counts, and visualized of cell types with t-SNE and UMAP (**Figure 1B**; **Supplementary Figures 1, 2**). We identified the immune cell types in all the groups, and the cell-type-specific canonical marker genes of different immune cells are displayed in **Supplementary Table 2** (**Figure 1C**). To analyze the heterogeneity of metadata, we carried out correlation analysis and PCA of all samples. Samples among different age groups showed obvious heterogeneity. The transcriptomic characteristics of G1 and G5 are significantly different from the other three groups, while the samples of G2, G3, and G4 show more similar distribution. It was age that drives the clustering of samples, rather than other heterogeneity such as gender (**Supplementary Figure 3**). The percentages of CD3^+^CD4^+^ T cells and CD3^+^CD8^+^ T cells in PBMCs at the scRNA-seq level were consistent with those measured by flow cytometry using canonical markers (**Supplementary Figures 4A, B**).

### The proportion and molecular characteristics of monocytes change significantly from G1 to G2.

**Figure 2:**
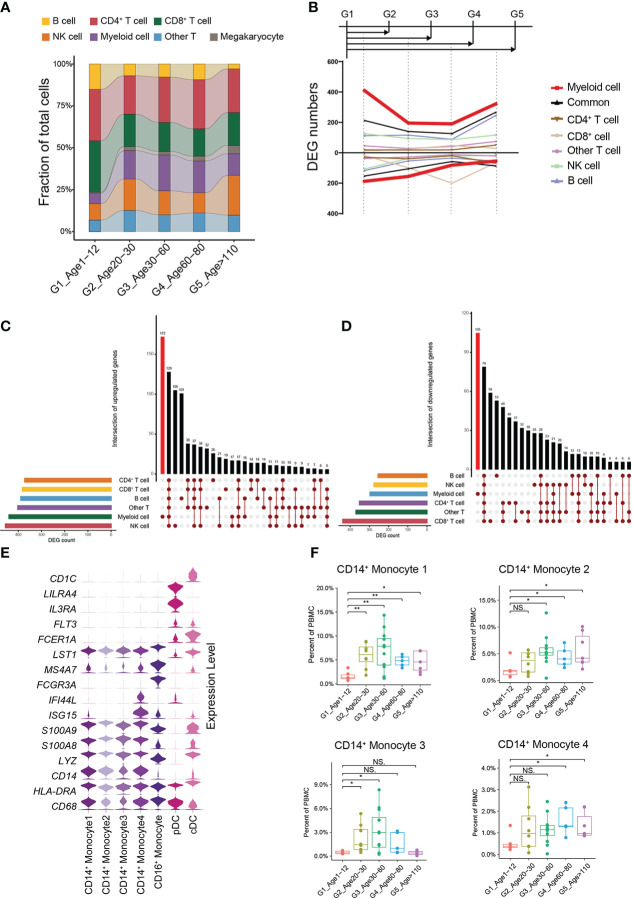
Changes in cellular proportion and molecular characteristics with age. **(A)** Composition of the main cell types in the 5 age groups. **(B)** Smoothed line plot displaying the number of specific and common DEGs of different cell types for pairwise comparisons with a G1 reference. Positive (negative) values represent upregulated (downregulated) genes. **(C)** UpSet plot showing the integrated comparative analysis of upregulated DEGs in the main cell types between G1 and the other groups. Upregulated DEGs: upregulated in G1, downregulated in other groups. **(D)** UpSet plot showing the integrated comparative analysis of downregulated DEGs in the main cell types between G1 and the other groups. Downregulated DEGs: downregulated in G1, upregulated in other groups. **(E)** Violin plots showing the expression distribution of selected canonical cell markers in the 7 subtypes of myeloid cells. **(F)** Boxplots of the percentage of 4 CD14^+^ monocyte subtypes in PBMCs. All differences with P < 0.01 are indicated. NS, not statistically significant; *P < 0.01; **P < 0.001.

In general, G1 had an increased percentage of B cells and a decreased percentage of myeloid cells compared to the other groups (**Figure 2A**). Moreover, the percentage of B cells gradually decreased with age, while the percentage of myeloid cells gradually increased with age (**Figure 2A**). We found that this trend began as G1 to G2. What is not the same as a change in the proportion of immune cells is a change in the molecular characteristics of immune cells. We therefore performed pairwise comparison of differential expression in each other groups relative to G1, and found that the number of cell-specific differentially expressed genes (DEGs) in myeloid cells was the most obvious (**Figure 2B**, Methods). At the same time, the number of DEGs in myeloid cells was the highest when G1 was compared with G2. On the other hand, we performed an integrated comparative analysis of DEGs of immune cells between G1 and other groups and found that myeloid cells had the highest number of cell-specific DEGs (**Figures 2C, D**). Myeloid cells included 4 groups of CD14^+^ monocytes, CD16^+^ monocytes, plasmacytoid dendritic cells (pDCs) and classic dendritic cells (cDC) (**Figure 2E**) (**Supplementary Table 2**). Among them, the percentages of all CD14^+^ monocyte subtypes in G1 were significantly lower than those in the other groups (**Figure 2F**; **Supplementary Figure 4C**). By analyzing the specific DEGs of different myeloid cell subtypes in G1, we found that different CD14^+^ monocyte subtypes showed heterogeneous transcriptional characteristics. The CD14^+^ monocyte 4 subtype was mainly enriched in interferon-related genes (**Figure 2E**), while the top enriched GO terms of the CD14^+^ monocyte 1, 2, and 3 subtypes were enriched in catabolic process, regulation of binding and response to cytokine, and T cell activation and T cell differentiation in thymus, respectively (**Supplementary Figures 3D–F**). By constructing a multi-age PBMC profile, we found that the cell percentage and molecular characteristics of myeloid cells were significantly altered from G1.

### A new subtype of CD8^+^ T cells specific to children

**Figure 3:**
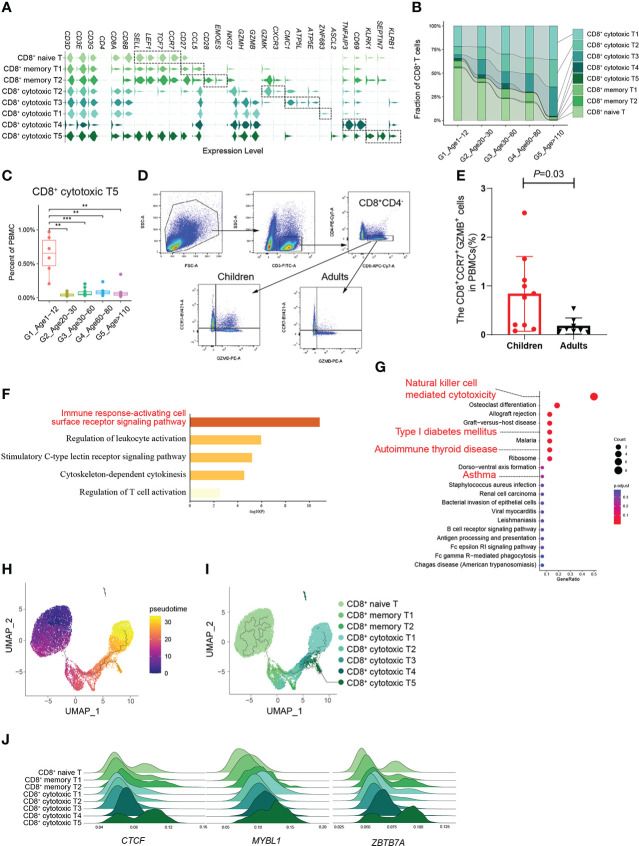
A distinct subtype of CD8^+^ cytotoxic T cells in childhood. **(A)** Violin plots showing the expression distribution of selected canonical cell markers in the 8 subtypes of CD8^+^ T cells. **(B)** Composition of CD8^+^ T cells in the 5 age groups. **(C)** Boxplots of the percentage of CD8^+^ cytotoxic T5 subtype in PBMCs. All differences with *P* < 0.01 are indicated. **P < 0.001; ***P < 0.0001. **(D)** The gating strategy of CD8^+^CCR7^+^GZMB^+^ T cells analyzed by flow cytometry. **(E)** The scatter plot showed the proportion of CD8^+^CCR7^+^GZMB^+^ T cells in PBMCs of healthy children and healthy adults (P=0.03). Differences with *P* < 0.05 are indicated. **(F, G)** Results of GO enrichment analysis **(F)** and KEGG pathway enrichment analysis **(G)** of the top50 marker genes of CD8^+^ cytotoxic T5. **(H)** Pseudotime trajectory of CD8^+^ T cells in G1 estimated using Monocle 3. **(I)** Pseudotime trajectory of CD8^+^ T cells in each group estimated using Monocle 3. **(J)** Transcription factors unique to the CD8^+^ cytotoxic T5 subtype.

According to the expression of canonical marker genes (**Supplementary Table 2**), there were 8 CD8^+^ T cell subtypes: naive CD8^+^ T cells, 2 subtypes of memory CD8^+^ T cells (CD8^+^ memory T1, T2) and 5 subtypes of cytotoxic CD8^+^ T cells (CD8^+^ cytotoxic T1~T5) (**Figure 3A**). It should be noted that the proportion of naive CD8^+^ T cells decreased significantly with age, whereas that of cytotoxic CD8^+^ T cells increased significantly (**Figure 3B**; **Supplementary Figures 5A, B**). In addition, the CD8^+^ cytotoxic T2 subtype, with high *GZMK* expression and low *GZMB* expression, was significantly increased in G5, which was consistent with a recent study revealing the relationship between GZMK^+^GZMB^-^CD8^+^T cells and aging (5) (**Supplementary Figures 5C, D**). Subsequently, we found that the percentage of the CD8^+^ cytotoxic T5 subtype was significantly higher in childhood than in other age groups, especially in other groups that were very rare (**Figure 3C**). By analyzing the gene signatures of the CD8^+^ cytotoxic T5 subtype, we found that the CD8^+^ cytotoxic T5 subtype significantly expressed both naive marker genes (*SELL*, *LEF1*, *TCF7*, and *CCR7*) and cytotoxic marker genes (*NKG7*, *GZMH*, and *GZMB*) (**Figure 3A**). Based on the molecular characteristic of the CD8+ cytotoxic T5 cell subtype, five protein markers (CD3, CD4, CD8, CCR7, GZMB) were used to analyze CD8^+^CCR7^+^GZMB^+^ T cells by flow cytometry. We performed flow cytometry analysis on the peripheral blood of children and adults, and found that the proportion of CD8^+^CCR7^+^GZMB^+^ T cells in children was significantly higher than that in adults (*P*=0.03) (**Figures 3D, E**; **Supplementary Figures 6A, B**).

### Molecular characteristics of CD8^+^CCR7^+^GZMB^+^ T cells

Given their unclear biological functions, we further focused on the molecular characteristics of the CD8^+^ cytotoxic T5 cell subtype that express both naive and cytotoxic marker genes. GO enrichment analysis and PPI analysis were performed on the top 50 specific genes of CD8^+^ cytotoxic T5 cell subtype (**Supplementary Table 4**), and they are mainly related to immune response-activating cell surface receptor signaling pathway and regulation of leukocyte activation (**Figure 3F**), and *SEPTIN*-related genes (*SEPTIN* 1,6,7,9) may play a key role in it (**Supplementary Figure 6C**). Septins are evolutionarily conserved in their crucial role in cytokinesis and tune actomyosin forces during motility and probably regulate lymphocyte trafficking in confined tissues (17, 18). Similarly, KEGG analysis found that it is related to T1D, autoimmune thyroid disease and asthma which were AIDs (**Figure 3G**). In addition, we found CD8^+^ cytotoxic T5 cell subtype had significantly expressed *ASCL2* and *KLRB1* (CD161) (**Figure 3A**; **Supplementary Figure 6D**). Ectopic expression of *Ascl2* downregulated *CCR7* expression in T cells *in vitro*, as well as accelerating T cell migration to the follicles and TFH-cell development *in vivo* in mice (19). However, both *CCR7* and *ASCL2* were significantly expressed in the CD8^+^ cytotoxic T5 cell subtypes (**Figure 3A**). Recent evidence suggested that CD8^+^CD161^+^ T cells were effector memory cells with stem cell characteristics that upregulate granzyme B and perforin and become highly cytotoxic upon activation (20). We performed pseudotime analysis of CD8^+^ T cells in G1 and found that the CD8^+^ cytotoxic T5 subtype had a unique differentiation trajectory. (**Figures 3H, I**; **Supplementary Figure 6E**). In addition, we identified some transcription factors that are more prominent in the CD8^+^ cytotoxic T5 subtype, such as *CTCF*, *MYBL1*, and *ZBTB7A* (**Figure 3J**). These results indicated that the CD8^+^ cytotoxic T5 subtype was a group of CD8^+^ cytotoxic T cells different from the others, which might play a special function in the childhood immune environment.

### Molecular characterization of B cells and CD4^+^ T cells in multiple age groups

The effect of age change on peripheral blood immune cells involves multiple cell types.

**Figure 4:**
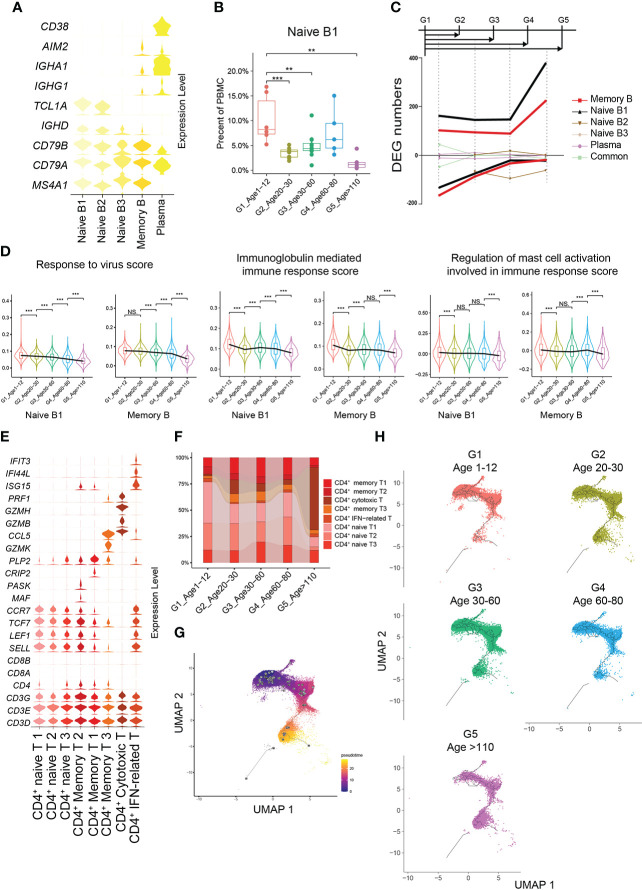
Molecular characterization of B cells and CD4^+^ T cells in multiple age groups. **(A)** Violin plots showing the expression distribution of selected canonical cell markers in the 5 subtypes of B cells. **(B)** Boxplots of the percentage of naive B1 subtype in PBMCs. All differences with P < 0.01 are indicated. NS, not statistically significant; **P < 0.001; ***P < 0.0001. **(C)** Smoothed line plot displaying the number of specific and common DEGs of different B cell subtypes for pairwise comparisons with a G1 reference. Positive (negative) values represent upregulated (downregulated) genes. **(D)** Expression levels of 3 GO biological process terms in naive B1 and memory B subtypes across the 5 age groups. All differences with *P* < 0.01 are indicated. **P < 0.001; ***P < 0.0001. **(E)** Violin plots showing the expression distribution of selected canonical cell markers in the 8 subtypes of CD4^+^ T cells. **(F)** Composition of CD4^+^ T cells in the 5 age groups. **(G)** Pseudotime trajectory of CD4^+^ T cells estimated using Monocle 3. **(H)** Pseudotime trajectory of CD4^+^ T cells in each group estimated using Monocle 3.

G1 had the highest percentages of naive B1 and naive B2 subtypes compared with the other groups (**Figures 4A, B**). The number of cell-specific DEGs of naive B1 and memory B cell subtypes was significantly higher than that in other B cell subtypes (**Figure 4C**). In G1, the naive B1 subtype specifically upregulated functions related to the response to virus and mast cell activation involved in the immune response, while the memory B subtype upregulated functions related to antigen processing and presentation, immunoglobulin-mediated immune response and viral processes (**Supplementary Figures 7A, B**). Hence, we evaluated the expression levels of the gene set related to these important functions in naive B1 and memory B subtypes across all age groups. Naive B1 and memory B subtypes in G1 had higher scores for functionally relevant gene sets for “response to virus”, “immunoglobulin mediated immune response” and “regulation of mast cell activation involved in immune response” than other age groups (**Figure 4D**, Methods). The age-related reduction in naive CD4^+^ T cells is consistent with CD8^+^ T cells (**Figures 4E, F**; **Supplementary Figure 7C**). Previous studies found a high level of CD4^+^ cytotoxic T cells in G5 (21), which paralleled our results (**Supplementary Figure 7C**). As expected, the pseudotime results of CD4^+^ T cells formed a transcriptional continuum, ranging from naive to cytotoxic CD4^+^ T cells (**Figure 4G**; **Supplementary Figures 7E, F**). At the same time, different age groups also exhibited unique pseudotime distributions (**Figure 4H**).

### Abundant ligand–receptor interactions of PBMCs in childhood

**Figure 5:**
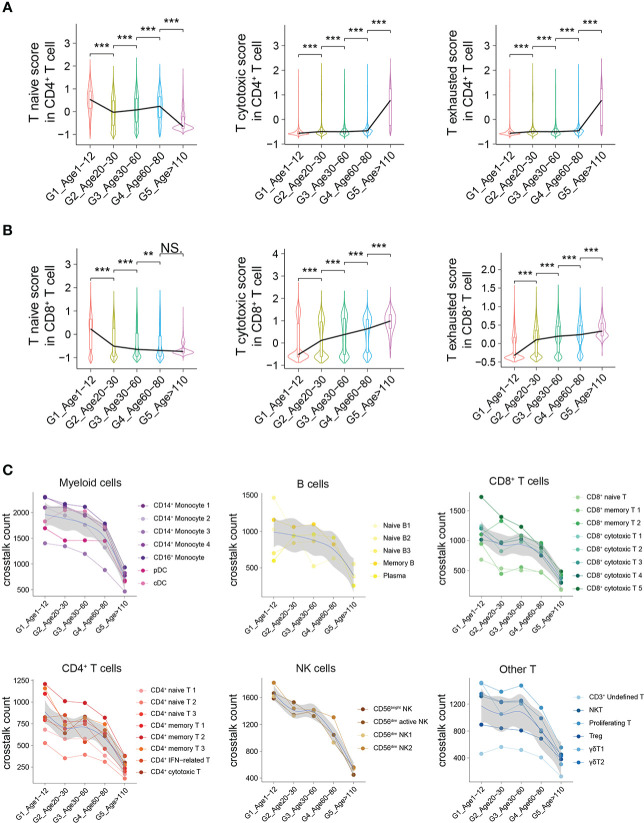
Active intercellular crosstalk of PBMCs in childhood. **(A, B)** Naive state, cytotoxicity and exhaustion scores of different CD4^+^ T cells **(A)** and CD8^+^ T cells across 5 groups. All differences with *P* < 0.01 are indicated. NS, not statistically significant; **P < 0.001; ***P < 0.0001. **(C)** The intercellular crosstalk number of each main cell type estimated using Cellphone DB, including myeloid cells, B cells, CD8^+^ T cells, CD4^+^ T cells, NK cells and other T cells.

To further demonstrate the characteristics of PBMCs with age, we comprehensively evaluated the functions and states of 4 main cell types of PBMCs (myeloid cells, CD4^+^ T cells, CD8^+^ T cells and B cells) across multiple age groups. The functional scores of “immune effector process”, “activation of immune response”, “establishment or maintenance of cell polarity”, “regulation of defense response” and “leukocyte activation” in all main cell types were significantly decreased with age (**Supplementary Figures 8A–E**). However, the functional scores of “leukocyte homeostasis”, “cytokine production involved in immune response” and “leukocyte migration” in all main cell types increased first and then decreased, with the increase of age (**Supplementary Figures 8F–H**). For CD4^+^ T cells, the naive score in G1 was significantly higher than that in the other groups, while the scores of “cytotoxicity” and “exhaustion” in G1 were significantly lower than those in the other groups (**Figure 5A**). In contrast, the naive score and cytotoxic score of CD8^+^ T cells in G1 were significantly higher than those in the other groups, whereas the exhaustion score was significantly lower than that in the other groups (**Figure 5B**). In addition, through comparing multiple age groups, we found that CD8^+^ T cells showed significant changes in the early stage (G1 to G2), whether “naive score”, “cytotoxic score” or “exhaustion score”, while CD4^+^ T cells changed in the late stage (G4 to G5).

The number of ligand–receptor interactions between each cell type gradually decreased with age (**Figure 5C**). Nevertheless, the number of ligand–receptor interactions of different cell types had different trends according to aging, such as myeloid cells and B cells with a slower decline and NK cells with a rapid decline (**Figure 5C**). Taking different monocyte subtypes as an example, the number of ligand–receptor interactions in each cell subtype also decreased significantly with age (**Supplementary Figure 9**). Thus, we believe that crosstalk between PBMCs is very frequent in childhood, which may be one of the characteristics of the immune system in childhood.

### Cell-specific distribution of susceptibility genes for AIDs

**Figure 6:**
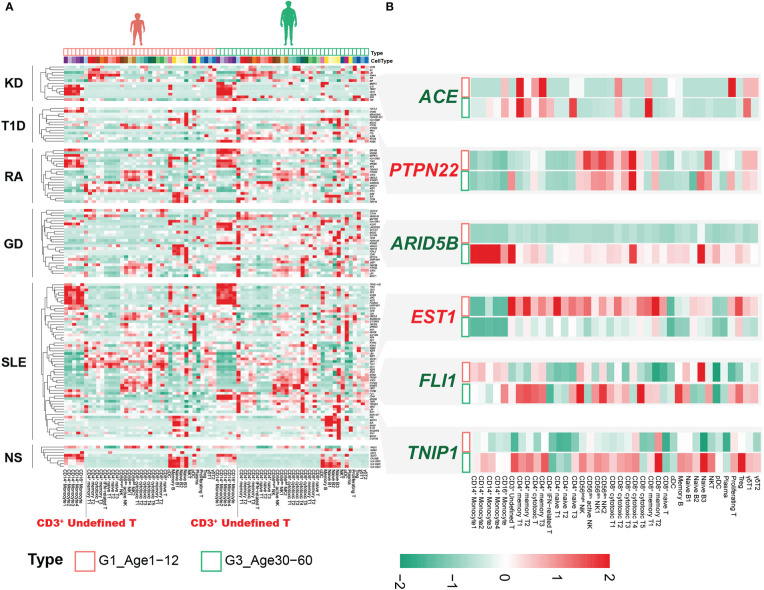
Combined analysis of genes associated with immune-related disease risk. **(A)** Heatmap of susceptibility genes for AIDs in G1 and G3. **(B)** Susceptibility genes with significantly different expression between G1 and G3.

There are significant differences in the autoimmune diseases (AIDs) spectrum of different ages (22–25), which may be related to the molecular characteristics of PBMCs at different ages (26, 27). GWASs have successfully identified thousands of disease-associated variants, and single-cell profiles allow the construction of multiple gene programs to relate GWAS variants more finely to function (28). We assessed the expression levels of susceptibility genes reported in GWASs of AIDs in PBMCs, including Kawasaki disease (KD), type 1 diabetes mellitus (T1D), rheumatoid arthritis (RA), Graves’ disease (GD), systemic lupus erythematosus (SLE) and nephrotic syndrome (NS). Sex differences in immune cells have been well documented (29), with G1 being close to G3 in sex ratio (**Supplementary Figure 1B**, **Supplementary Table 6**), and we contrasted the differences between these two age groups. For KD, the expression of susceptibility genes was mainly distributed in monocytes, CD4^+^ T cells and γδ T cells, especially in monocytes (**Figure 6A**). Susceptibility genes for RA and SLE could be divided into 5 groups, including monocytes, CD4^+^ T cells, CD8^+^ cytotoxic cells, NK cells and B cells (**Figure 6A**). Nephrotic syndrome is a group of diseases that may be associated with immune disorders, leading to kidney podocyte injury and thus proteinuria (30). Its susceptibility genes are mainly expressed in monocytes and B cells. Although there are several HLA-related genes in the susceptibility genes of nephrotic syndrome, we can clearly see that the susceptibility genes are significantly expressed in B cells (**Figure 6A**). This may provide clues for B cell therapy in nephrotic syndrome, such as rituximab targeting CD20 (31).

Notably, the CD3^+^ undefined T cells clearly expressed susceptibility genes for multiple AIDs, especially in G3 (**Figure 6A**). Although not statistically significant, we observed that the CD3^+^ undefined T cells was elevated in G3 (**Supplementary Figure 10A**). DEGs analysis of CD3^+^ undefined T cells between G1 and G3 showed that it significantly down-regulated *TNFSF13B*, *ARID5B* and up-regulated *IGKC*, *SEPTIN7* and other genes in G1 (**Supplementary Figure 10B**). CD3^+^ undefined T cells expressed the marker genes of CD3^+^ naive cells, but did not express B cell-related marker genes (**Supplementary Figure 10C**). Transcriptional factor analysis revealed a pattern of transcriptional regulation in CD3^+^ undefined T cells that was similar to CD8^+^ cytotoxic T4 and Naive B3 (**Supplementary Figure 10D**). However, some transcription factors, such as *FOXB1*, *ZEB1*, *STAT3* and so on, were expressed prominently in CD3^+^ undefined T cells, making them different from other T cells (**Supplementary Figure 10D**). At the same time, by comparing the cross-talk between CD3^+^ undefined T cells in G1 and G3, we found that TNFSF13B related pathway was up-regulated in G3, and the ligand-receptor relationship of CCL5-CCR4 only existed in CD3^+^ undefined T cells in G3 (**Supplementary Figures 10E, F**). These results suggested that CD3^+^ undefined T cells may be a special type of immune cells in adult life.

### Susceptibility genes for AIDs were differentially expressed in different age groups

The expression of susceptibility genes in AIDs not only showed obvious cellular localization, but also changed between G1 and G3. The expression level of *ETS1* was extensively down-regulated in circulating immune cells in G3 (**Figure 6B**). The SNP (rs1128334) associated with SLE resulted in decreased *ETS1* expression (32), and down-regulation of *ETS1* expression was also found in PBMCs of SLE patients (33). Recent studies have confirmed that *ETS1* mainly involves CD4^+^ T cells to participate in the occurrence and development of SLE (34). We found that the down-regulation of *ETS1* expression was significant not only in CD4^+^ T cells, but also in CD8^+^ T cells (**Figure 6B**). However, it was unclear what effect *ETS1* has on CD8^+^ T cells. This suggested that the extensive down-regulation of *ETS1* expression in G3 may be associated with the pathogenesis of SLE. The chromatin accessibility of *ETS1* was regulated by the inhibitory effector T-cell transcription factor *FLI1*, whose loss increases the chromatin accessibility of *ETS1 (* 35). Interestingly, *FLI1* was significantly upregulated in CD4^+^ effector T cells and CD8^+^ effector T cells in G3 (**Figure 6B**), and previous studies confirmed that *FLI1* expression was significantly upregulated in SLE patients (36). These results emphasized that *FLI1* may be involved in the regulation of *ETS1* in CD4^+^ effector T cells and CD8^+^ effector T cells of SLE patients.

*ARID5B* associated with a variety of AIDs (37, 38), significantly up-regulated in multiple cell subsets of G3, especially in monocytes (**Figure 6B**). The expression of *ARID5B* in monocytes of the elderly (>65 years old) was significantly up-regulated, which may be related to atherosclerosis (39). Our results indicated that changes in the expression of this gene may occur earlier (30-60 years old) and may be common to multiple immune cells. In addition, we also found that other susceptibility genes related to AIDs were differentially expressed in different age groups, such as *ACE*, *PTPN22*, *TNIP1*, etc. (**Figure 6B**).

## Discussion

Based on single-cell transcriptome sequencing data, we constructed a PBMC profile over multiple age groups, ranging from 1 year old to over 110 years. We comprehensively displayed the functional characteristics of PBMCs in childhood at the single-cell level, including the percentage of cell subtypes, gene expression differences, ligand–receptor relationships and pseudotime relationships. In contrast to previous reference studies on the percentage of immune cells in childhood (13–15), using as little as 2 mL of blood as material, we analyze all major immune cell populations by high-throughput method, providing the landscape of immune system in childhood. Given the influence of ethnic factors on the immune characteristics of different age groups, only East Asian populations were selected for our research in different age groups. At the same time, the correlation analysis and PCA among the samples suggested that the samples of different age groups showed significant heterogeneity. Age was the main factor driving the sample clustering, while there was no obvious clustering for different genders (**Supplementary Figure 3**). Significant changes in immune cells during embryonic and neonatal periods have been demonstrated (40, 41); thus, our study did not include these specific age groups for comparison.

Previous studies have found that the percentage of circulating monocytes progressively increases with age (42) and circulating monocytes show similar transcriptional signatures in young and elderly healthy individuals (43). We found significant time-dependent changes in both the number and the gene expression levels of monocytes, with significant changes occurring at an early stage (from G1 to G2). In addition, different CD14^+^ monocyte cell subtypes had different time-dependent changes, and future research on changes in monocytes with age may require attention to distinguish between different CD14^+^ monocyte subtypes (**Figure 2E**). Furthermore, we found that the CD4^+^ cytotoxic T cell subtype and the GZMK^+^GZMB^-^CD8^+^ T cell subtype were significantly expanded in supercentenarians; the expansion of the former was believed to be one of the reasons why supercentenarians are less susceptible to chronic diseases and tumors (21), and the latter was thought to be the senescence-associated CD8^+^ T cell subtype (5).

We considered that the significant differential changes in PBMCs from G1 to G2 might be closely related to the degeneration of the thymus during puberty and the changes in the development of myeloid and lymphoid cells in the bone marrow. We also showed the dynamic changes in the ratio of naive and effector types in CD4^+^ T cells and CD8^+^ T cells. More researches are needed to explore the possible mechanisms by why CD8^+^ T cells changed significantly at the early stage (G1 to G2) but CD4^+^ T cells changed only at the late stage (G4 to G5), and the possible role of this differential change throughout the life cycle. At the same time, we innovatively found that the CD8^+^CCR7^+^GZMB^+^ cytotoxic T cell subtype might be a group of circulating immune cells specific to childhood, and this cell subtype expressed both naive and cytotoxic T cell marker genes. The specific genes and characteristic functions of CD8^+^CCR7^+^GZMB^+^T cells indicated a special function for this niche cell population. The CD8^+^CCR7^+^GZMB^+^ cytotoxic T cell subtype that significantly expressing *KLRB1* could have similar cellular functions to CD8^+^CD161^+^ T cells (20), but more evidence is needed.

We also demonstrated changes in the crosstalk of PBMCs at multiple ages, and the cell–cell communication of PBMCs is evident in childhood. Genome-wide association studies have identified many disease-related susceptibility genes (44). Combined with the genomics results, we found that susceptibility genes for AIDs showed cell specificity in our immune cell profile (**Figure 6**). Meanwhile, the cell type-specific expression of susceptibility genes varied in different AIDs, which suggested the key contribution of the associated cell types in the disease process. Surprisingly, we found that B cells in nephrotic syndrome expressed multiple susceptibility genes (**Figure 6A**). Although many of these molecules are HLA-related, they also provide valuable clues for future immune-related studies in nephrotic syndrome. Our data indicated a possible relationship between CD3^+^ undefined T cells and AIDs and explored their molecular features (**Figure 6**; **Supplementary Figure 10**). However, we did not find the characteristic marker genes for this subpopulation, which may be due to the small number of cells. The upregulation of *TNFSF13B* expression in this cell subtype and its effect on the ligand-receptor relationship need to be determined in future studies.

In conclusion, our study displayed the molecular characteristics of PBMCs in childhood adults and centenarians at the single-cell level and provided new evidence to elucidate the special immune environment of different age groups. Due to the small sample size as one of the major shortcomings of this study, our results may not fully display the immune cell landscape in cross the lifespan, and future studies with larger cohort are needed (27). Sex bias existed in our multiple age groups, and future studies could observe the changes of immune system of different genders in different age groups. Although PBMCs are a window into the entire immune system (45), recent studies have found that organ senescence may be associated with immune cell infiltration in tissues and consequently lead to systemic inflammation (46). Although our work did not involve immune cells in various organs of children, we believe that more research will be carried out to reveal the differences in immune cells in tissues and organs of children and other age groups. Moreover, T cell receptor (TCR) and B-cell receptor (BCR) V(D)J transcriptome analysis at a single-cell resolution might be a powerful tool for exploring the origin and nature of lymphocytes in organs (47, 48), which was not conducted in our research. This research provided a valuable basis for future research on the unique immune system of childhood and AIDs.

## Data availability statement

The original contributions presented in the study are publicly available. We have uploaded the data of self-tested scRNA-seq to the GEO database (GSE206295).

## Ethics statement

The studies involving human participants were reviewed and approved by Institutional Review Board of Children’s Hospital of Chongqing Medical University. Written informed consent to participate in this study was provided by the participants’ legal guardian/next of kin.

## Author contributions

All authors contributed to the article and approved the submitted version. QC and QL conceived the study. JZ analyzed the data with assistance from RD, and wrote the manuscript. HJ, LL, XF, JW, MC, LP, XL and JL performed experiments and analyzed data. MW and HY reviewed the manuscript.

## Supplementary material

Supplementary Figure 1 scRNA-seq data quality assessment and processing. **(A)** The number of UMIs and genes, and the percentage of mitochondrial genes ribosomal genes for each of the donors in the 5 groups. **(B)** Gender composition of each age group. **(C–E)** Two-dimensional principal component analysis (PCA) **(C)**, UMPA **(D)** and tSNE **(E)** visualization of PBMCs for multiple age groups. Different colors represent 43 clusters (cell types) illustrated in tSNE plots. **(F)** Two-dimensional visualization of PBMCs for each group illustrated in UMPA (left) plots and tSNE plots (right).

Supplementary Figure 2 Two-dimensional UMAP visualization of PBMCs for each sample.

Supplementary Figure 3 Heterogeneity analysis of samples. **(A)** The heatmap showed the results of correlation analysis among the samples. **(B)** The PCA result of all samples at the bulk-level.

Supplementary Figure 4 Assessment of unpublished data and an integrated comparative analysis of the DEGs of the main immune cell types. **(A, B)** The flow cytometric analysis results of CD3^+^CD4^+^ T cells and CD3^+^CD8^+^ T cells in CON-2 and CON-3. **(C)** Boxplots of the percentage of CD16^+^ monocyte, cDC and pDC subtypes in PBMCs. All differences with *P* < 0.01 are indicated. *P < 0.01; **P < 0.001. **(D–F)** GO enrichment analyses of the specific Up-DEGs of CD14^+^ monocyte 1 **(D)**, 2 **(E)**, and 3 **(F)** subtypes in G1.

Supplementary Figure 5 Characterization of CD8^+^ T cells in childhood. **(A)** Boxplots of the percentage of each subtype of CD8^+^ T cells in PBMCs. All differences with *P* < 0.01 are indicated. *P < 0.01; **P < 0.001; ***P < 0.0001 **(B)** Boxplots of the percentage of CD8^+^ cytotoxic T in CD8^+^ T cells. All differences with *P* < 0.01 are indicated. *P < 0.01; **P < 0.001. **(C)** Boxplots of the percentage of CD8^+^ cytotoxic T2 in PBMCs. All differences with *P* < 0.01 are indicated. ***P* < 0.001. **(D)** The distributions of the CD8^+^ cytotoxic T2 subtype in CD8^+^ T cells in the G1 and G5 groups illustrated in UMAP plots.

Supplementary Figure 6 Characteristics of CD8^+^CCR7^+^GZMB^+^ T cells. **(A, B)** The proportion of CD8^+^CCR7^+^GZMB^+^ T cells to CD8^+^ T cells in peripheral blood of 10 healthy children **(A)** and 8 healthy adults **(B)**. **(C)** Results of PPI analysis of the top50 marker genes of CD8^+^ cytotoxic T5. **(D)** The gene expression level of *ASCL2* and *KLRB1* in different subtype of CD8^+^ T cells. All differences with *P* < 0.01 are indicated. NS, not statistically significant; ***P* < 0.001; ****P* < 0.0001. **(E)** Pseudotime trajectory of each CD8^+^ T cell subtypes in G1 estimated using Monocle 3.

Supplementary Figure 7 Characterization of B cells and CD4^+^ T cells in childhood. **(A, B)** GO enrichment analyses of the specific Up-DEGs of Naive B1 **(A)** and Memory B **(B)** subtypes in G1. **(C)** Boxplots of the percentage of CD4^+^ naive T1 and CD4^+^ cytotoxic T in PBMCs. All differences with *P* < 0.01 are indicated. *P < 0.01; **P < 0.001; ***P < 0.0001. **(D)** Smoothed line plot displaying the number of specific and common DEGs of different CD4^+^ T cell subtypes for pairwise comparisons with a G1 reference. Positive (negative) values represent upregulated (downregulated) genes. **(E)** Pseudotime trajectory of CD4^+^ T cells in G1 estimated using Monocle 3. A continuous value was assigned to each cell as a pseudotime. **(F)** Pseudotime trajectory of each CD4^+^ T cell subtypes in G1 estimated using Monocle 3.

Supplementary Figure 8 Functional differences in the main cell types across the 5 groups. **(A–H)** The functional scores of “immune effector process” (GO:0002252) **(A)**, “activation of immune response” (GO:0002253) **(B)**, “establishment or maintenance of cell polarity” (GO:0007163) **(C)**, “regulation of defense response” (GO:0031347) **(D)**, “leukocyte activation” (GO:0045321) **(E)**, “leukocyte homeostasis” (GO:0001776) **(F)**, “cytokine production involved in immune response” (GO:0002367) **(G)**, and “leukocyte migration” (GO:0050900) **(H)** of the main cell types in the 5 groups. All differences with *P* < 0.01 are indicated. NS, not statistically significant; *P < 0.01; **P < 0.001; ***P < 0.0001.

Supplementary Figure 9 Active intercellular crosstalk of myeloid cells in childhood. The intercellular crosstalk number of each subtype of monocyte cells estimated using Cellphone DB, including CD14^+^ monocyte 1, CD14^+^ monocyte 2, CD14^+^ monocyte 3, CD14^+^ monocyte 4 and CD16^+^ monocyte.

Supplementary Figure 10 Characterization of CD3^+^ undefined T cells. **(A)** Boxplots of the percentage of CD3^+^ undefined T subtype in PBMCs. **(B)** The volcano map showed the DEGs in CD3^+^ undefined T of the G1 vs G3 groups. **(C)** Expression of canonical marker genes of T cells and B cells in CD3^+^ undefined T. **(D)** The results of transcription factor analysis for CD3^+^ undefined T. **(E, F)** Dot plot showing the ligand–receptor relationship of the CD3^+^ undefined T subtype in G1 **(E)** and G3 **(F)**.
