# Supplementary figures and images for: Single-cell RNA sequencing reveals the molecular features of peripheral blood immune cells in children, adults and centenarians
Source: Front Immunol. 2023 Jan 10;13:1081889. doi: 10.3389/fimmu.2022.1081889 (PMC9871912; doi:10.3389/fimmu.2022.1081889)

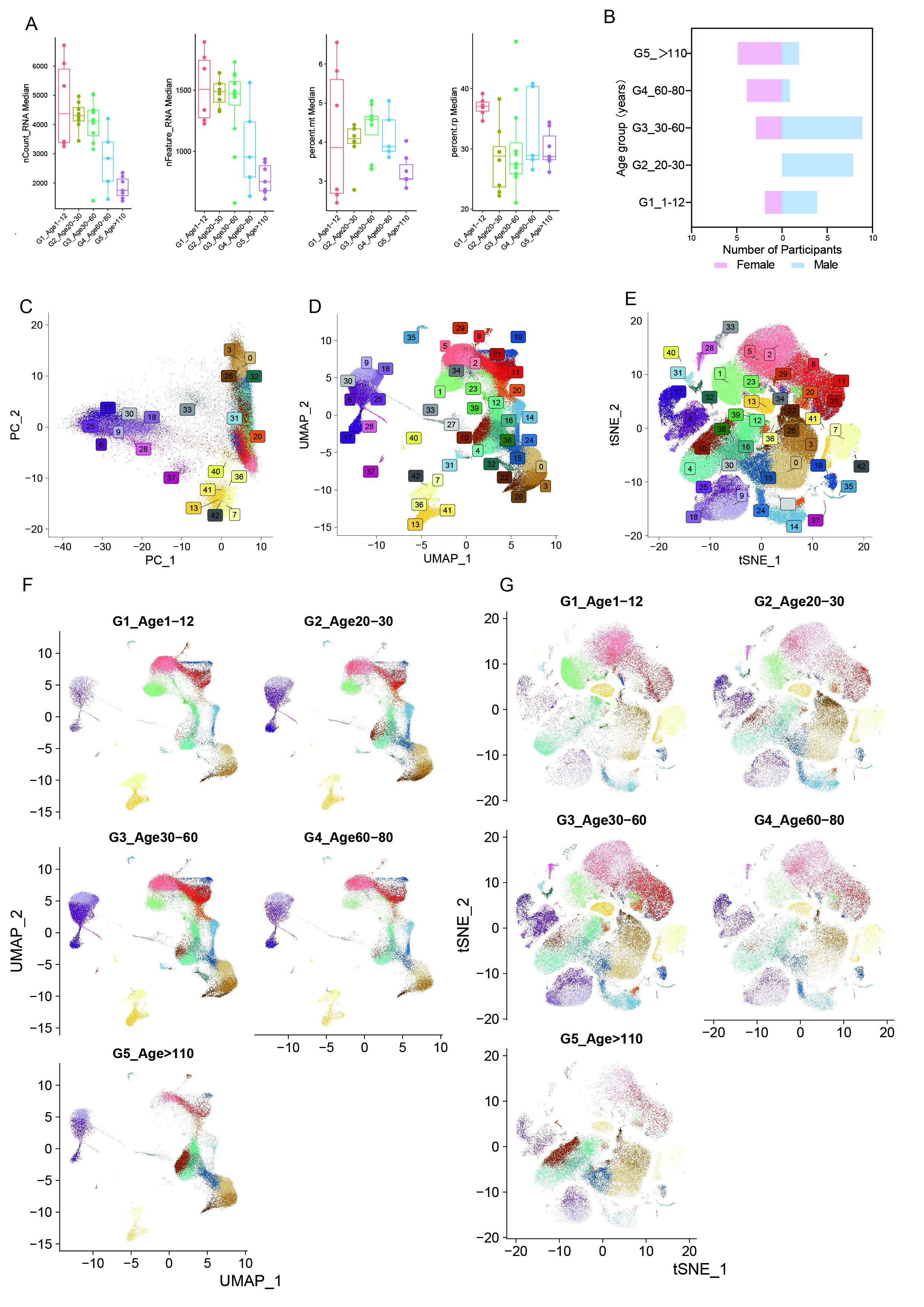

Supplement: Supplementary Figure 1 — scRNA-seq data quality assessment and processing. (A) The number of UMIs and genes, and the percentage of mitochondrial genes ribosomal genes for each of the donors in the 5 groups. (B) Gender composition of each age group. (C–E) Two-dimensional principal component analysis (PCA) (C), UMPA (D) and tSNE (E) visualization of PBMCs for multiple age groups. Different colors represent 43 clusters (cell types) illustrated in tSNE plots. (F) Two-dimensional visualization of PBMCs for each group illustrated in UMPA (left) plots and tSNE plots (right). [file Image_1.jpeg]

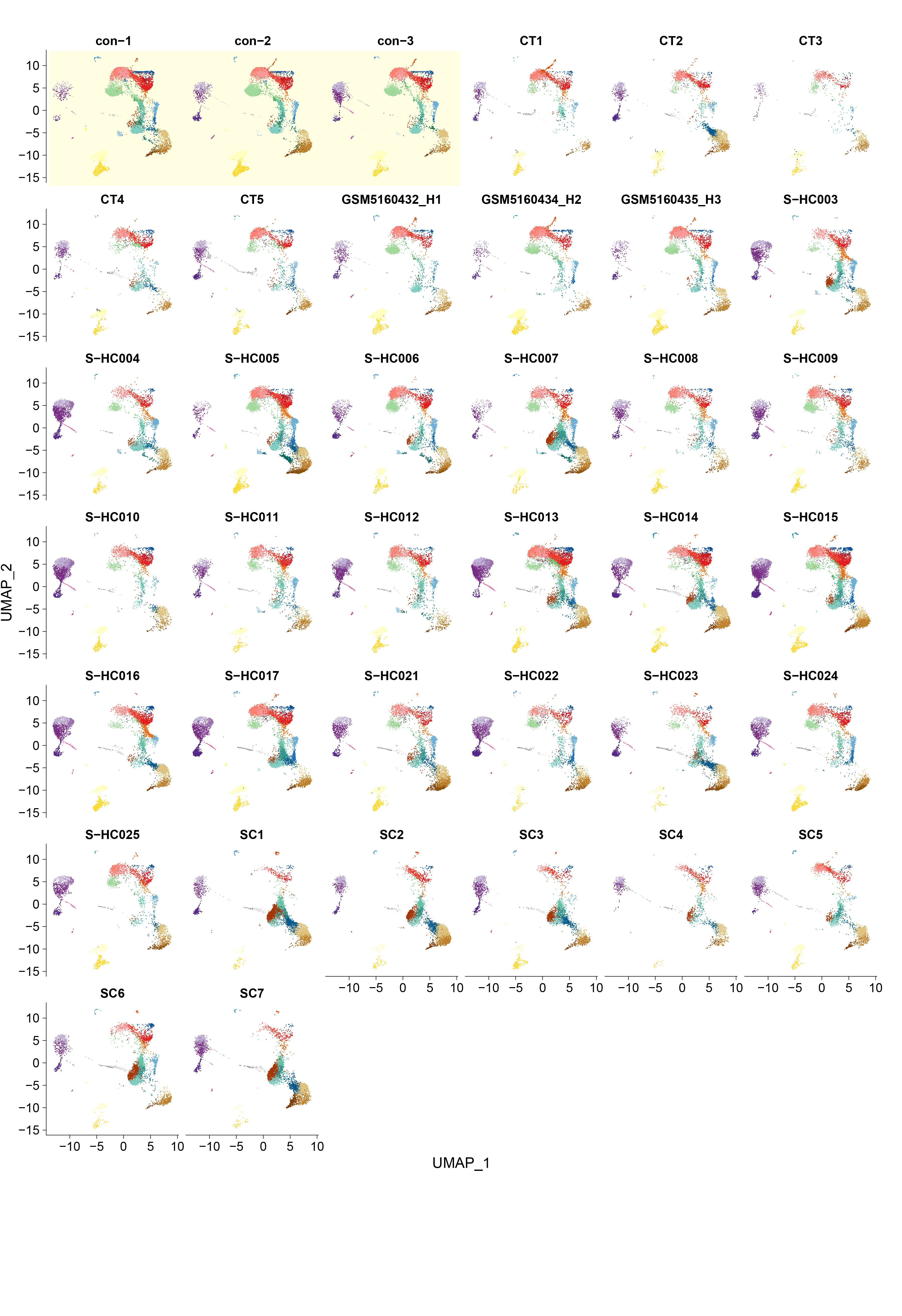

Supplement: Supplementary Figure 2 — Two-dimensional UMAP visualization of PBMCs for each sample. [file Image_2.jpeg]

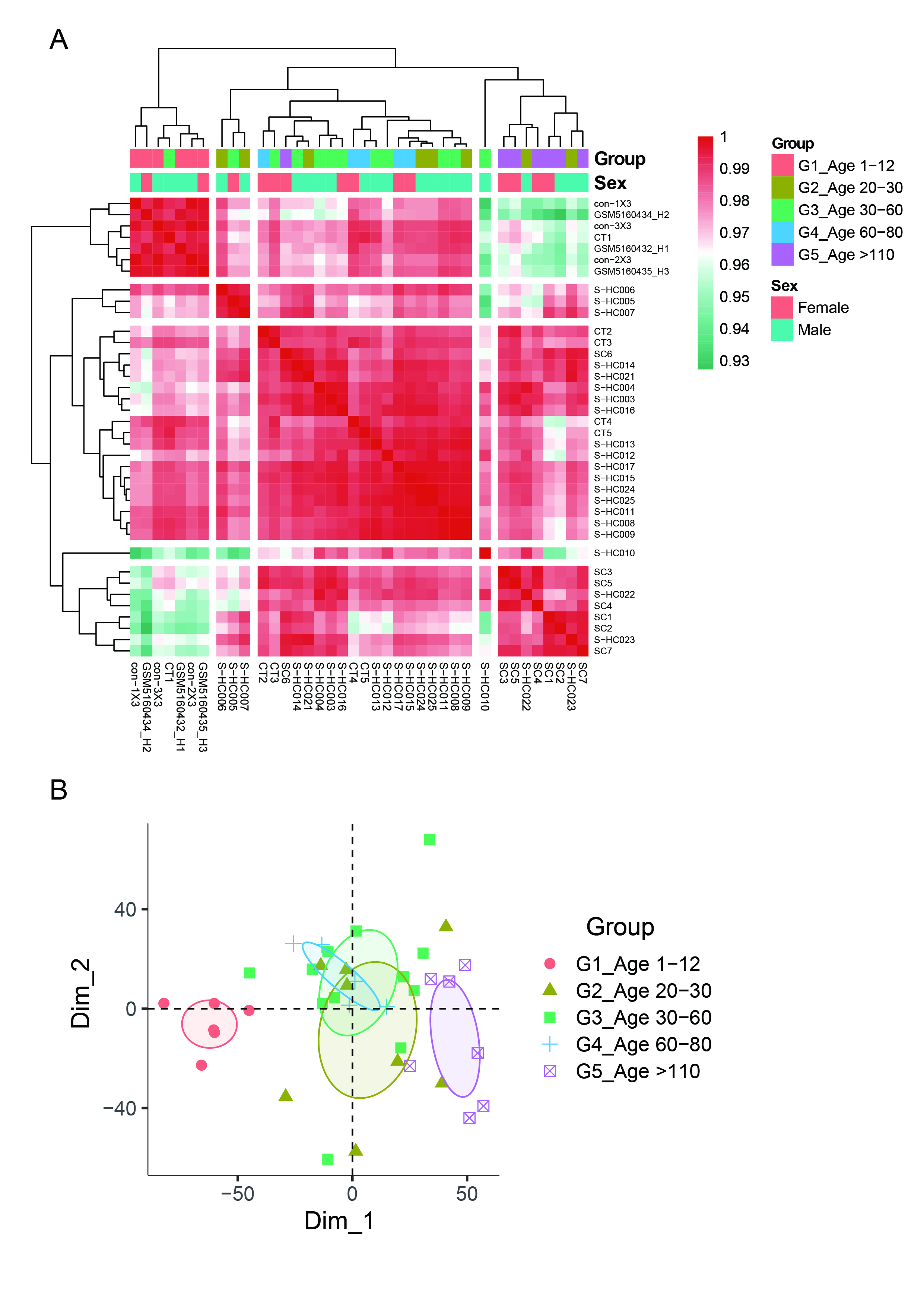

Supplement: Supplementary Figure 3 — Heterogeneity analysis of samples. (A) The heatmap showed the results of correlation analysis among the samples. (B) The PCA result of all samples at the bulk-level. [file Image_3.jpeg]

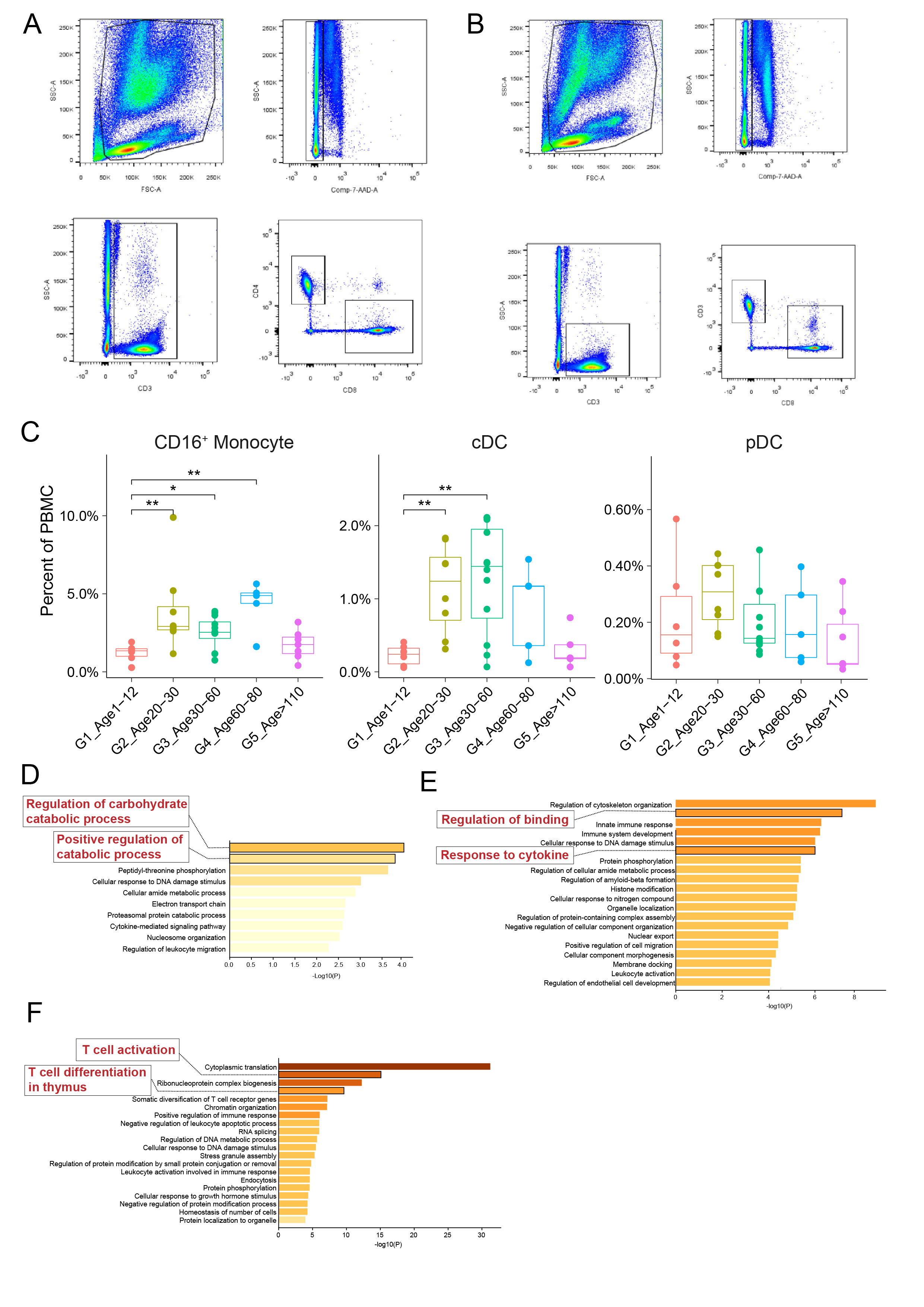

Supplement: Supplementary Figure 4 — Assessment of unpublished data and an integrated comparative analysis of the DEGs of the main immune cell types. (A, B) The flow cytometric analysis results of CD3+CD4+ T cells and CD3+CD8+ T cells in CON-2 and CON-3. (C) Boxplots of the percentage of CD16+ monocyte, cDC and pDC subtypes in PBMCs. All differences with P < 0.01 are indicated. *P < 0.01; **P < 0.001. (D–F) GO enrichment analyses of the specific Up-DEGs of CD14+ monocyte 1 (D), 2 (E), and 3 (F) subtypes in G1. [file Image_4.jpeg]

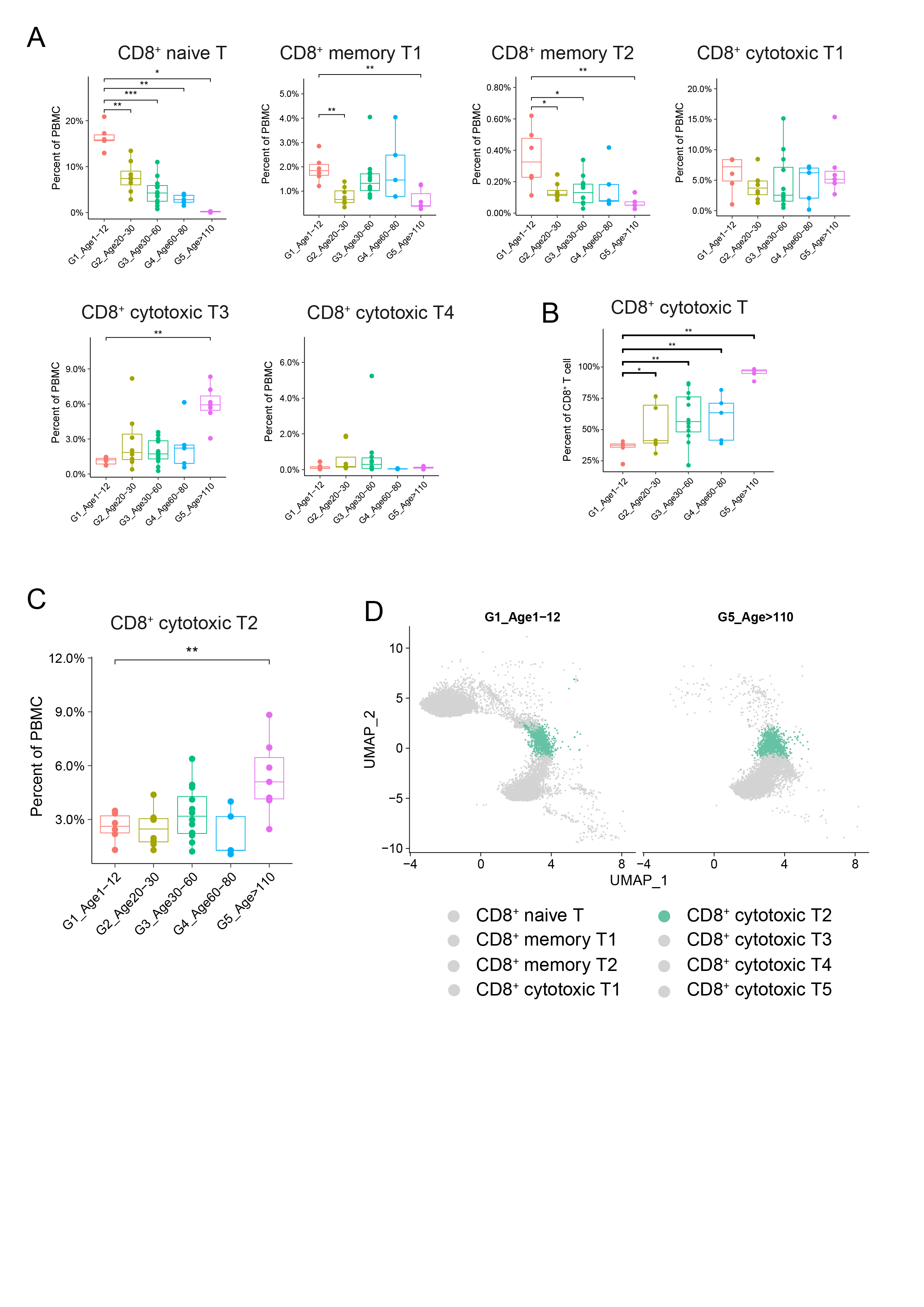

Supplement: Supplementary Figure 5 — Characterization of CD8+ T cells in childhood. (A) Boxplots of the percentage of each subtype of CD8+ T cells in PBMCs. All differences with P < 0.01 are indicated. *P < 0.01; **P < 0.001; ***P < 0.0001 (B) Boxplots of the percentage of CD8+ cytotoxic T in CD8+ T cells. All differences with P < 0.01 are indicated. *P < 0.01; **P < 0.001. (C) Boxplots of the percentage of CD8+ cytotoxic T2 in PBMCs. All differences with P < 0.01 are indicated. **P < 0.001. (D) The distributions of the CD8+ cytotoxic T2 subtype in CD8+ T cells in the G1 and G5 groups illustrated in UMAP plots. [file Image_5.jpeg]

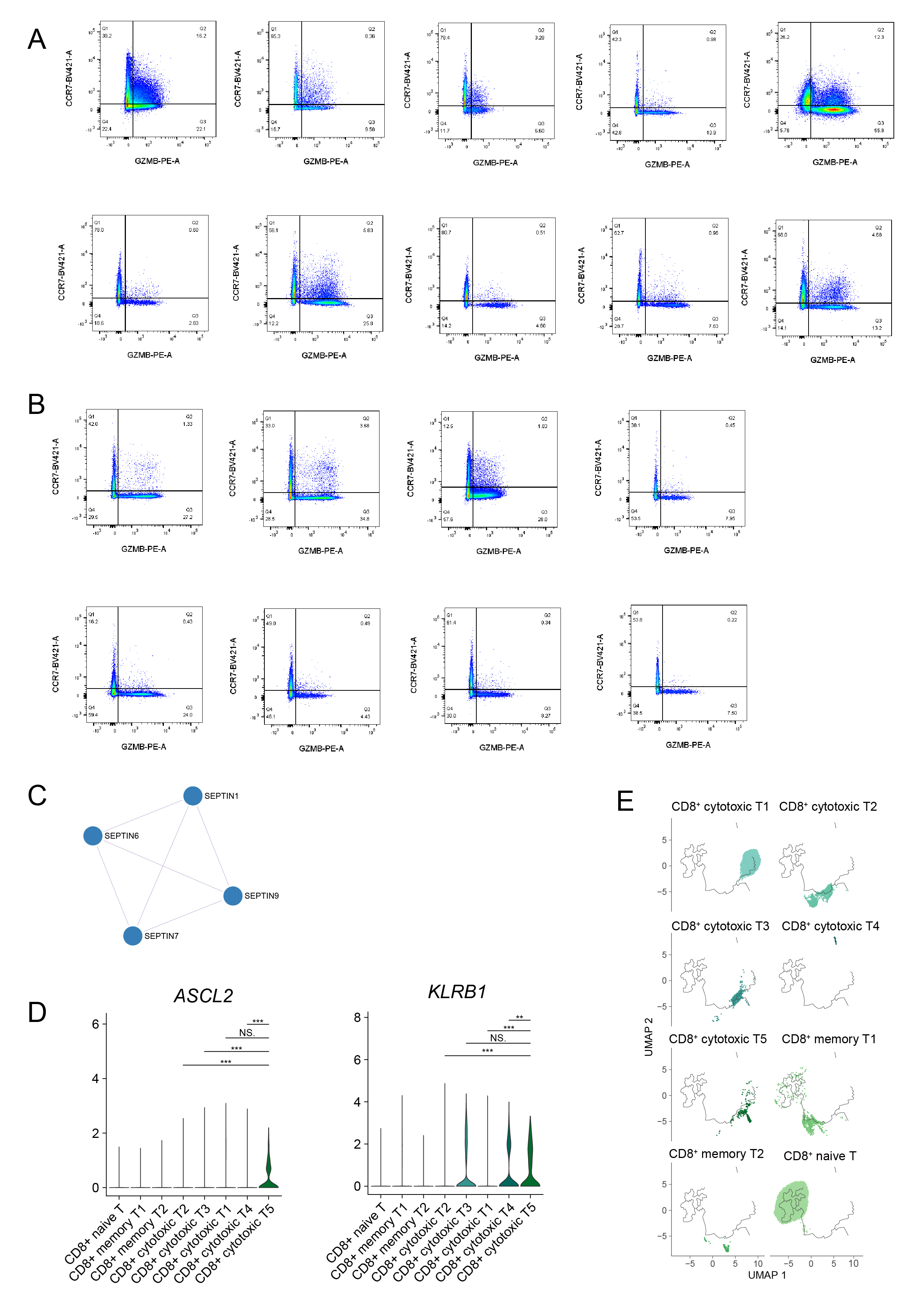

Supplement: Supplementary Figure 6 — Characteristics of CD8+CCR7+GZMB+ T cells. (A, B) The proportion of CD8+CCR7+GZMB+ T cells to CD8+ T cells in peripheral blood of 10 healthy children (A) and 8 healthy adults (B). (C) Results of PPI analysis of the top50 marker genes of CD8+ cytotoxic T5. (D) The gene expression level of ASCL2 and KLRB1 in different subtype of CD8+ T cells. All differences with P < 0.01 are indicated. NS, not statistically significant; **P < 0.001; ***P < 0.0001. (E) Pseudotime trajectory of each CD8+ T cell subtypes in G1 estimated using Monocle 3. [file Image_6.jpeg]

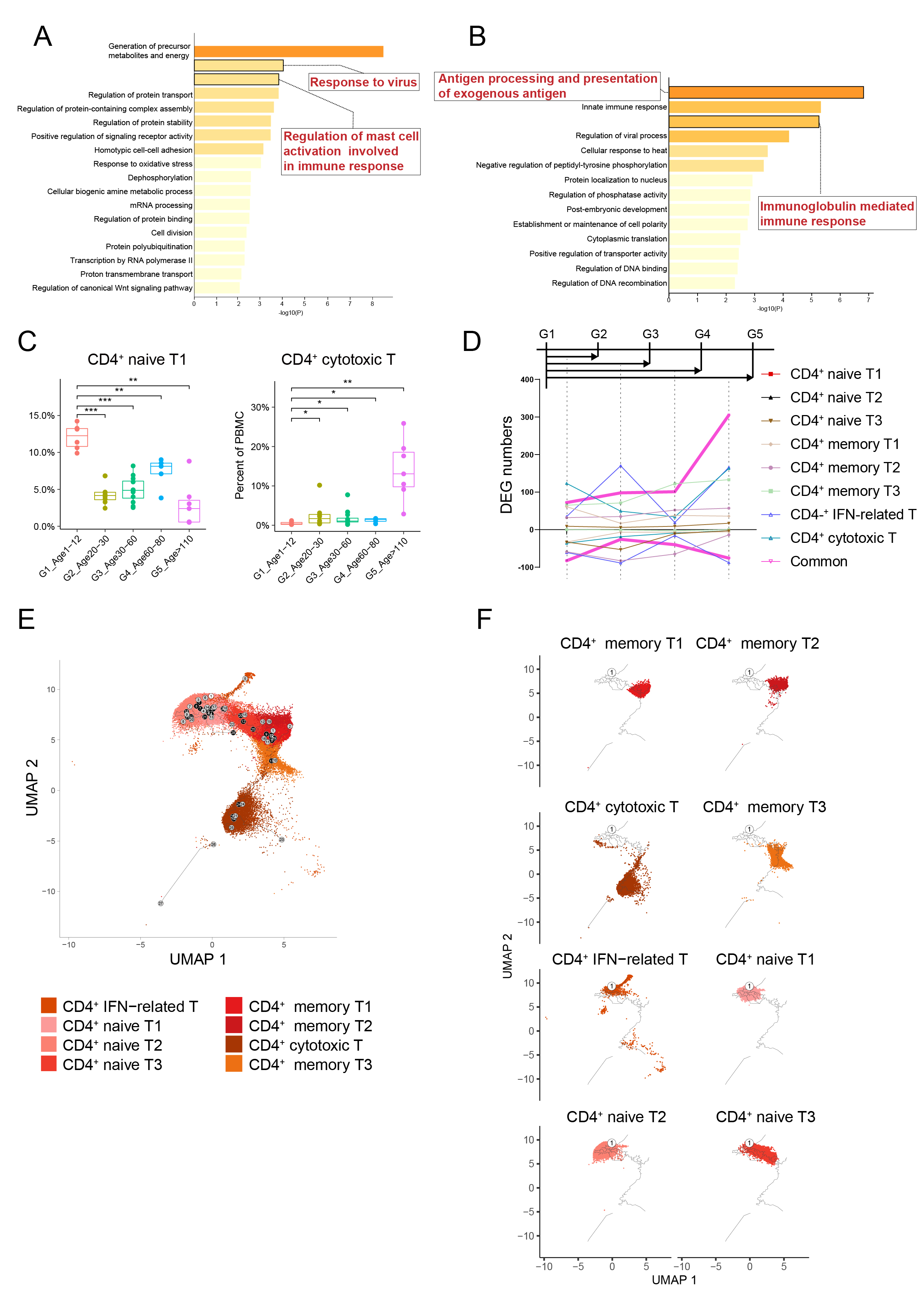

Supplement: Supplementary Figure 7 — Characterization of B cells and CD4+ T cells in childhood. (A, B) GO enrichment analyses of the specific Up-DEGs of Naive B1 (A) and Memory B (B) subtypes in G1. (C) Boxplots of the percentage of CD4+ naive T1 and CD4+ cytotoxic T in PBMCs. All differences with P < 0.01 are indicated. *P < 0.01; **P < 0.001; ***P < 0.0001. (D) Smoothed line plot displaying the number of specific and common DEGs of different CD4+ T cell subtypes for pairwise comparisons with a G1 reference. Positive (negative) values represent upregulated (downregulated) genes. (E) Pseudotime trajectory of CD4+ T cells in G1 estimated using Monocle 3. A continuous value was assigned to each cell as a pseudotime. (F) Pseudotime trajectory of each CD4+ T cell subtypes in G1 estimated using Monocle 3. [file Image_7.jpeg]

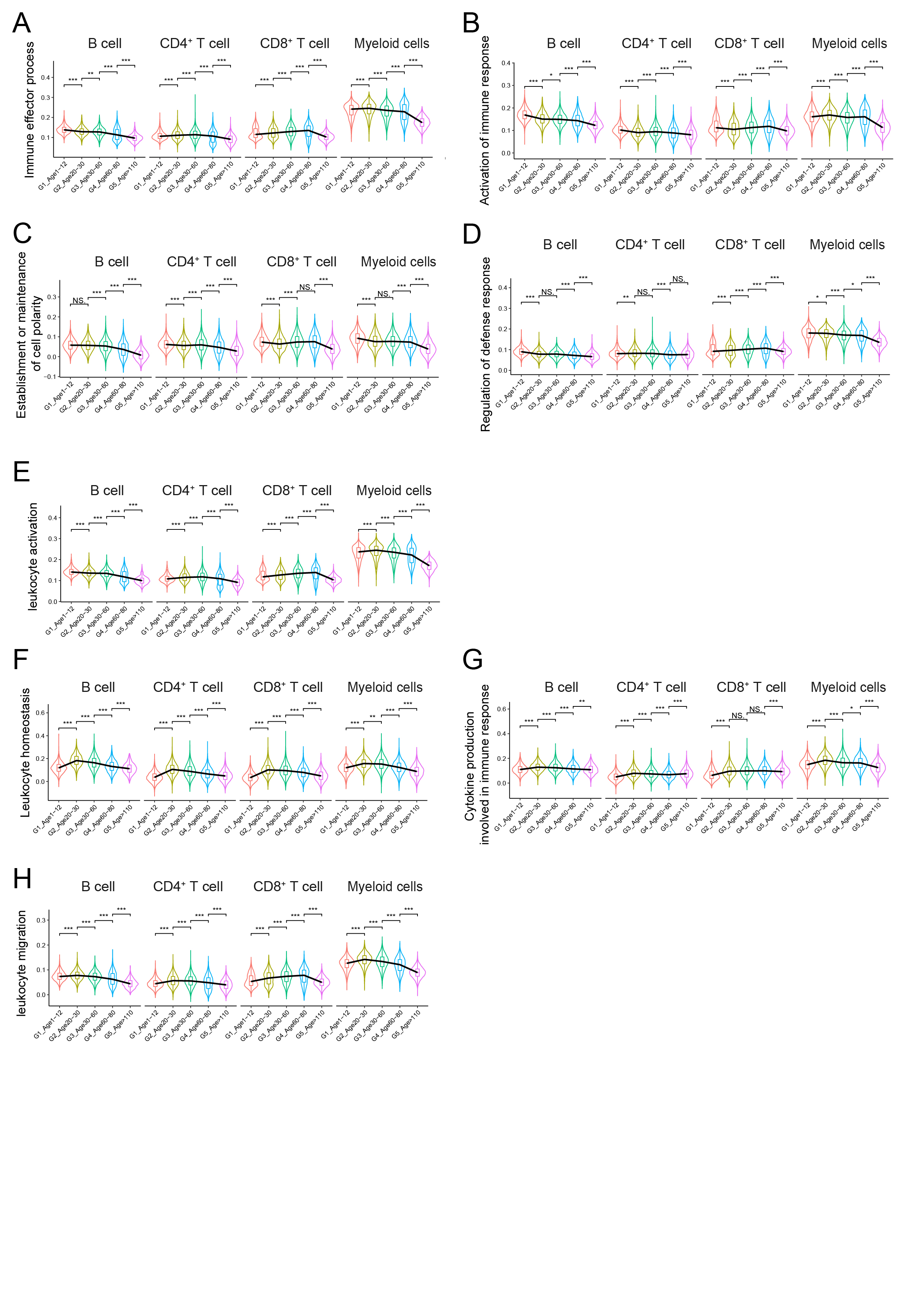

Supplement: Supplementary Figure 8 — Functional differences in the main cell types across the 5 groups. (A–H) The functional scores of “immune effector process” (GO:0002252) (A), “activation of immune response” (GO:0002253) (B), “establishment or maintenance of cell polarity” (GO:0007163) (C), “regulation of defense response” (GO:0031347) (D), “leukocyte activation” (GO:0045321) (E), “leukocyte homeostasis” (GO:0001776) (F), “cytokine production involved in immune response” (GO:0002367) (G), and “leukocyte migration” (GO:0050900) (H) of the main cell types in the 5 groups. All differences with P < 0.01 are indicated. NS, not statistically significant; *P < 0.01; **P < 0.001; ***P < 0.0001. [file Image_8.jpeg]

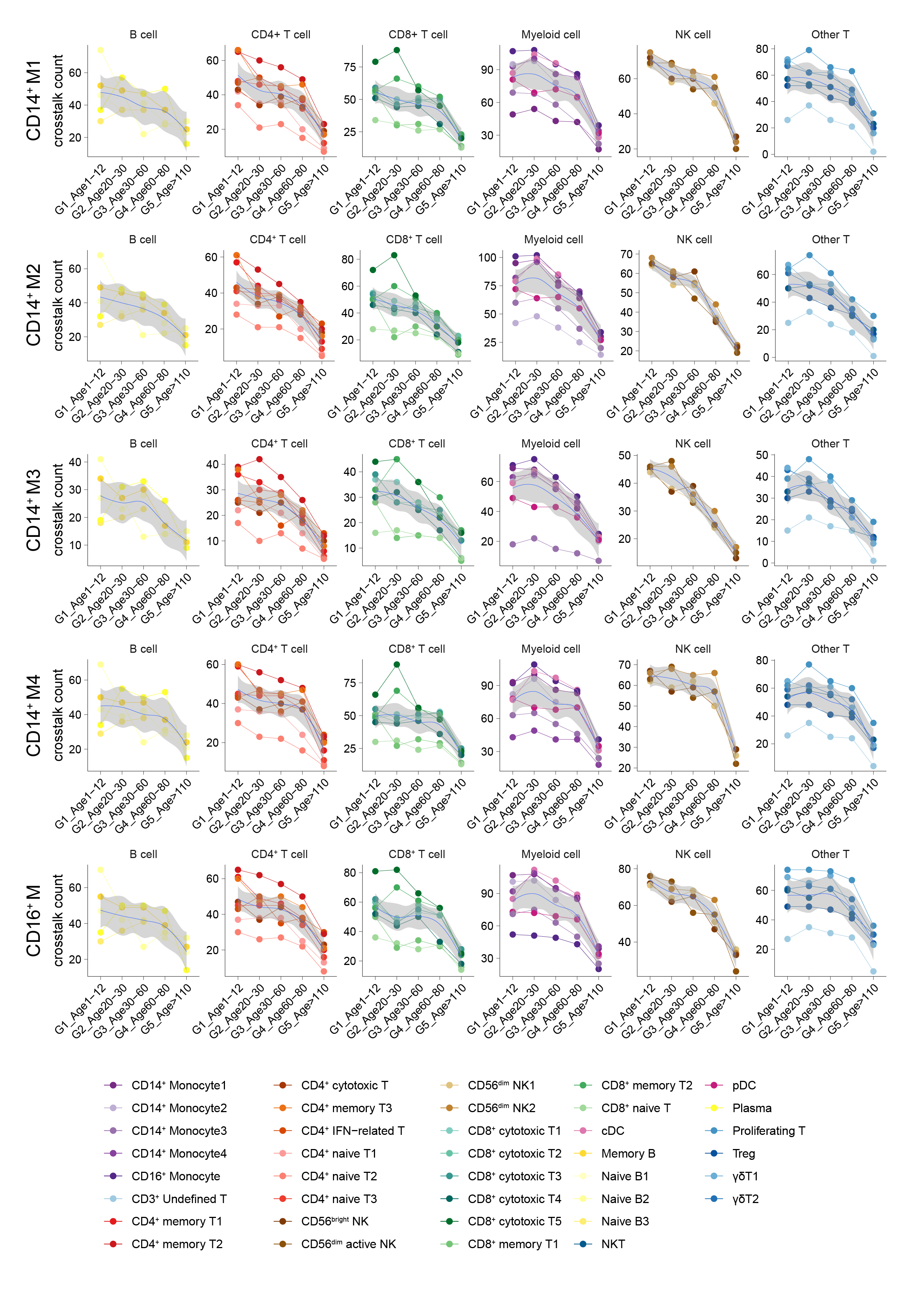

Supplement: Supplementary Figure 9 — Active intercellular crosstalk of myeloid cells in childhood. The intercellular crosstalk number of each subtype of monocyte cells estimated using Cellphone DB, including CD14+ monocyte 1, CD14+ monocyte 2, CD14+ monocyte 3, CD14+ monocyte 4 and CD16+ monocyte. [file Image_9.jpeg]

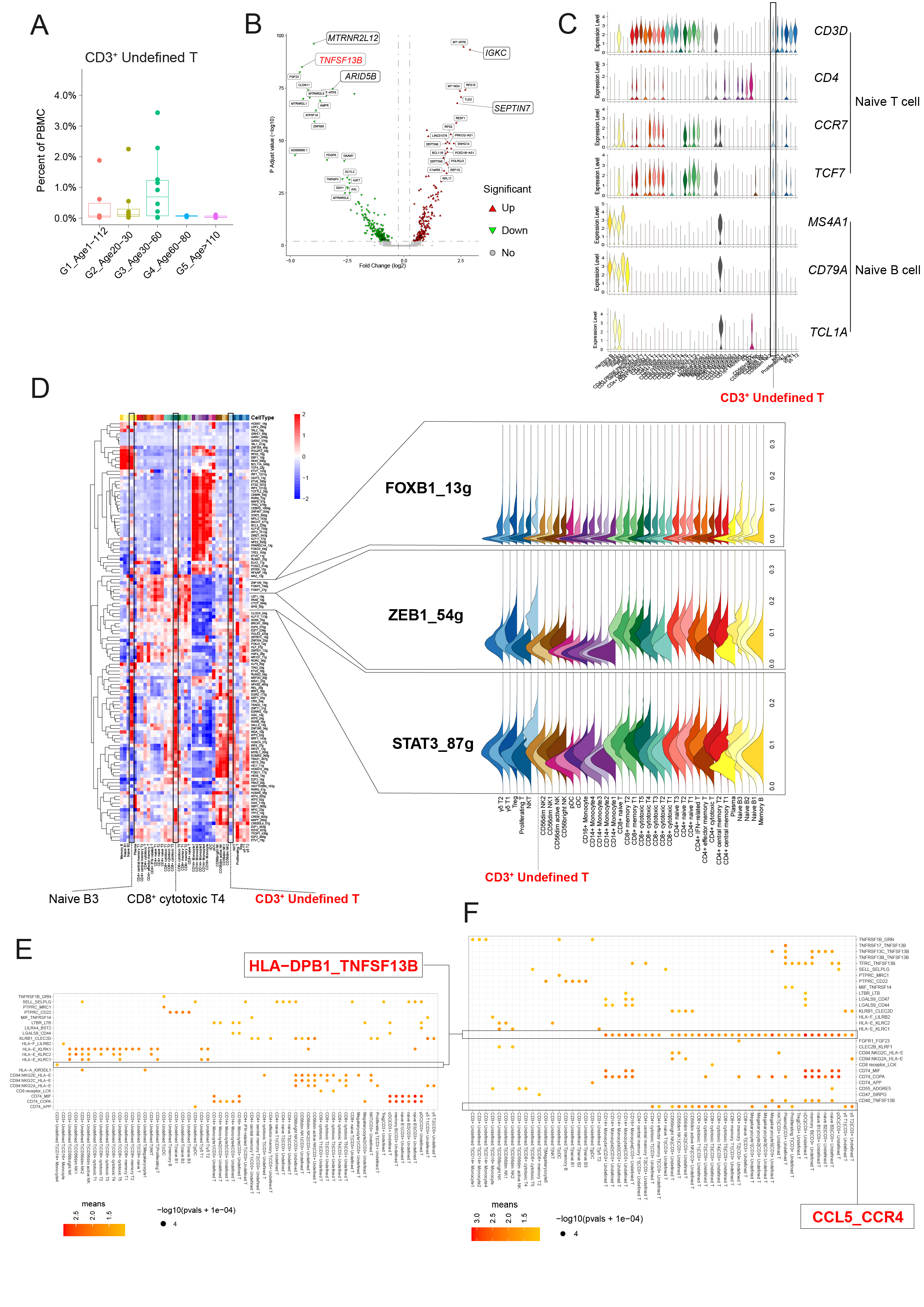

Supplement: Supplementary Figure 10 — Characterization of CD3+ undefined T cells. (A) Boxplots of the percentage of CD3+ undefined T subtype in PBMCs. (B) The volcano map showed the DEGs in CD3+ undefined T of the G1 vs G3 groups. (C) Expression of canonical marker genes of T cells and B cells in CD3+ undefined T. (D) The results of transcription factor analysis for CD3+ undefined T. (E, F) Dot plot showing the ligand–receptor relationship of the CD3+ undefined T subtype in G1 (E) and G3 (F). [file Image_10.jpeg]
